# Warmth and competence in your face! Visual encoding of stereotype content

**DOI:** 10.3389/fpsyg.2013.00386

**Published:** 2013-06-28

**Authors:** Roland Imhoff, Jonas Woelki, Sebastian Hanke, Ron Dotsch

**Affiliations:** ^1^Department Psychology, Social Psychology, Social Cognition, University of CologneCologne, Germany; ^2^Social and Legal Psychology, University of BonnBonn, Germany; ^3^Social and Cultural Psychology, Radboud University NijmegenNijmegen, Netherlands

**Keywords:** stereotypes content model, warmth, competence, reverse correlation, faces, visual representations

## Abstract

Previous research suggests that stereotypes about a group's warmth bias our visual representation of group members. Based on the stereotype content model (SCM) the current research explored whether the second big dimension of social perception, competence, is also reflected in visual stereotypes. To test this, participants created typical faces for groups either high in warmth and low in competence (male nursery teachers) or vice versa (managers) in a reverse correlation image classification task, which allows for the visualization of stereotypes without any a priori assumptions about relevant dimensions. In support of the independent encoding of both SCM dimensions hypotheses-blind raters judged the resulting visualizations of nursery teachers as warmer but less competent than the resulting image for managers, even when statistically controlling for judgments on one dimension. People thus seem to use facial cues indicating both relevant dimensions to make sense of social groups in a parsimonious, non-verbal and spontaneous manner.

What do we see when we mentally visualize a group member? Previous research suggests that our visual representation of group members is biased by the prejudice we hold against the group (Dotsch et al., 2008): individuals with stronger implicit prejudice against Moroccans expected typical Moroccan faces to look more criminal and less trustworthy. It may be highly efficient to visually encode information and knowledge about groups in terms of facial representations, because it offers an ecologically congruent and non-verbal mode of storing relevant information [see McArthur and Baron (1983)], like whether specific groups are likely to hurt or harm (i.e., warmth). Stereotypes, however, also carry information about how skilled or competent other groups are (Fiske et al., 2002). Whereas one's own group is usually regarded as relatively high on both dimensions, many other groups are seen as lacking either warmth (e.g., Asians, feminists, rich people), or competence (e.g., elderly, housewives, disabled), or both (welfare recipients, homeless). The identification of these two dimensions as central to social perception is not unique to the SCM. In fact, many different terminologies have been proposed to refer to two fundamental dimensions that seem to tap into comparable spheres, among others: morality and competence (Wojciszke et al., 1998), communion and agency (Bakan, 1956), or other-profitable and self-profitable traits (Peeters, 1983). Warmth and competence are thus deemed fundamental dimensions of social judgment (Abele et al., 2008). Here, we tested whether both fundamental dimensions of stereotypes (warmth and competence) are reflected in individuals' visual representations of group members.

At present, it is unclear whether knowledge about a group's position on each of the two dimensions is encoded in facial representations. Although warmth-related traits (i.e., trustworthiness or criminality) seem to be encoded in facial representations of social groups (Dotsch et al., 2008, 2013) this is less clear for competence. Previous research that purposefully addressed both dimensions has done so on a verbal level only, thereby ignoring any visual components. This is not only unfortunate because outside of psychological laboratories not group labels or any other verbal descriptors, but faces are the most frequent social stimuli we encounter (McArthur and Baron, 1983; Macrae and Quadflieg, 2010), but also because most of these tasks are inherently reactive and not spontaneous. Specifically, participants who are asked to report a group's position on a dimension or who complete reaction time tasks such as the Implicit Association Test (e.g., Carlsson and Björklund, 2010), are *forced* to evaluate groups on the respective dimension, barring any conclusions about spontaneous use of information on that dimension.

As reported above, warmth information seems to be spontaneously encoded in visual representations of groups, which raises the question whether the same can be concluded about competence information. The answer to this question does not necessarily parallel the findings with regards to warmth: it is conceivable that in spontaneous processing warmth information might be accessed and visually encoded more readily than competence information. This possibility seems to be corroborated by the fact that warmth is processed earlier (Abele and Bruckmüller, 2011), faster (Ybarra et al., 2001), more reliably (Willis and Todorov, 2006), and bears more weight in global impressions (Wojciszke et al., 1998) than competence. In the present work, we gave participants the opportunity to construe a group image without any constraints, enabling us to assess which dimensions are spontaneously accessed when thinking about groups on a visual level.

## The present research

To test which components of stereotypes are encoded in visual representation we selected social groups that differed on both dimensions in opposite direction (i.e., represent ambivalent stereotypes) that are not confounded with any other visually encoded feature (like different sexes, ethnicities). Professions were chosen as groups that had less clear associations with visible features like ethnicity, sex or age but for which previous research has shown that shared visual stereotypes exist (Hills et al., 2008; Oldmeadow et al., 2013). We pretested two occupation groups as ambivalent stereotyped groups (one high in warmth but low in competence, the other vice versa). Independent participants were then instructed to create a visualization of a typical face for a member of the respective occupation group to get an estimate of what individuals encode in visual group stereotypes. To test whether both stereotype dimensions were included we used two strategies, first, independent raters were asked to judge the resulting faces on both dimensions, assuming that any systematic effect can only be due to a successful decoding of previously encoded information. Second, we used objective physical similarity to visualizations of warm or competent faces.

## Methods

### Pretest: selecting ambivalent stereotypes

To study the visual encoding of both stereotype dimensions we selected the domain of stereotypes concerning professions. Fourteen professional groups that were assumed to map on the two ambivalent quadrants of the SCM (high warmth, low competence; low warmth, high competence) were selected and complemented with one group that was assumed to be high on both dimensions (physician) and one group that was assumed to be low on both dimensions (meter maid) to have an additional validity check. As our participants were German and professions (like most other nouns) are gendered in the German language we used separate lists for male and female representatives of the respective professions. These professional groups were then rated on both stereotype dimensions.

In an online study, 96 participants (57 women, 34 men, five without response; *M*_*age*_ = 29.86, *SD* = 10.51) rated all professional groups on warmth (benevolence, trustworthiness, heartiness; Cronbach's α ranged from 0.69 to 0.85, average α = 0.76) and competence (capability, efficiency, competitiveness; Cronbach's α ranged from 0.57 to 0.89, average α = 0.82) on a scale from 1 to 10. The extent to which all raters agreed on these stereotypes was calculated with intra-class coefficients. Rater agreement was high for warmth ratings of male and female professionals, both *ICC*s = 0.99, as well as for the competence ratings of male, *ICC* = 0.98, and female professions, *ICC* = 0.96.

**Figure 1:**
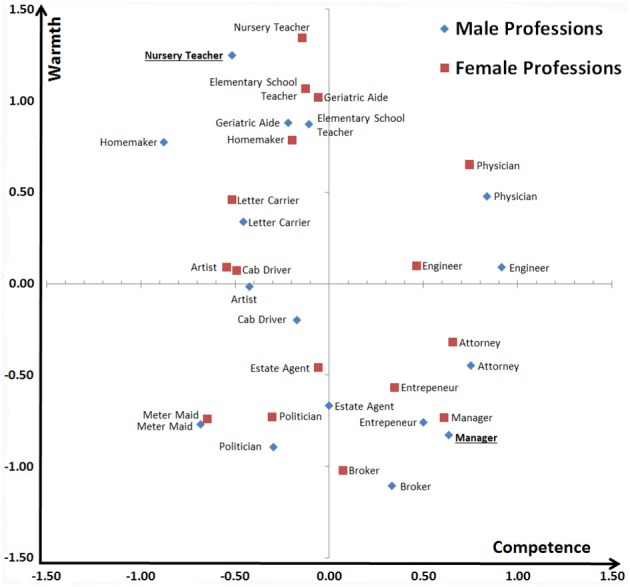
**Pretest ratings of warmth and competence (*z*-standardized means) for 32 professional groups**.

The mean values for each profession were then standardized by subtracting the grand mean across all 32 professions and divided by the standard deviation across all ratings. Figure 1 displays these *z*-standardized rating for all 32 groups. Based on an inspection of these results, male nursery teacher and male manger were chosen as the best exemplars of ambivalent stereotypes. Nursery teachers, *M* = 7.79, *SD* = 1.54, were clearly rated as warmer than managers, *M* = 3.63, *SD* = 1.65, *t*_(95)_ = 18.90, *p* < 0.001, *Cohen's d* = 1.93, and managers, *M* = 7.73, *SD* = 1.72, were seen as clearly more competent than nursery teachers, *M* = 5.82, *SD* = 1.81, *t*_(95)_ = 7.57, *p* < 0.001, *Cohen's d* = 0.78.

### Image construction

The selected professional groups were used in the image construction phase. Specifically, participants were randomly assigned to visualize either a male nursery teacher or a male manager. In addition, participants explicitly rated both groups on warmth and competence (explicit stereotypes) and completed an indirect measure of their association between the two groups and the concept of competence (implicit stereotypes).

#### Participants

For the image construction task, 92 new participants (80 women, 12 men; no nursery teachers or managers; *M*_*age*_ = 22.41, *SD* = 5.58) were recruited in the laboratory of the university. None of them had participated in the pretest.

#### Image creation

**Figure 2:**
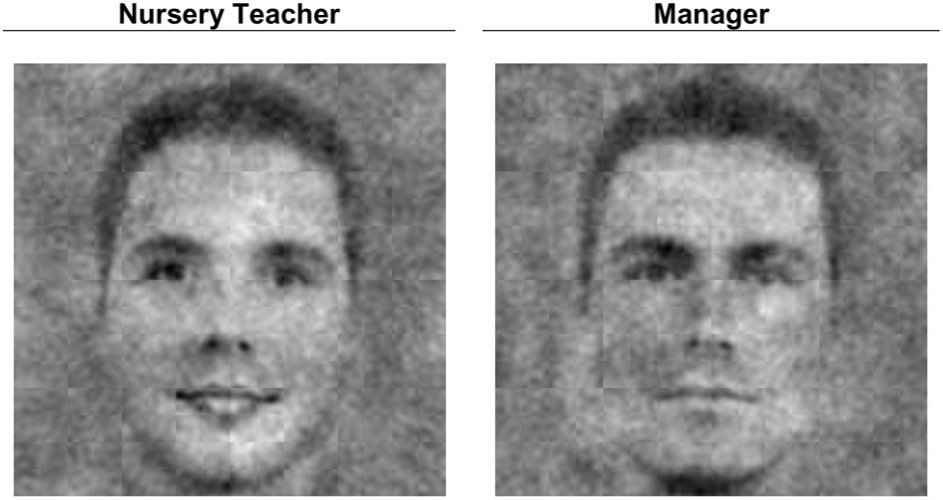
**Classification images for nursery teacher and manager based on responses of *N* = 92 (*n* = 46 per condition)**.

Visualizations of people's internal representation of the two respective professional groups were created with a two images forced choice reverse correlation image classification task (RCIC; Mangini and Biedermann, 2004; Dotsch et al., 2008; Imhoff et al., 2011). Half of the participants performed a nursery teacher RCIC task, whereas the other half performed a manager RCIC task. In 770 trials, participants picked from two faces presented simultaneously the face that looked more like a manager (or, in the other condition: nursery teacher). The faces were comprised of always the same base face (a 512 × 512 pixel low-pass filtered morph of 16 male faces; all faces were taken from the Radboud Face Database; Langner et al., 2010) and superimposed random visual noise (for noise generation, see Dotsch et al., 2008). For each condition, the average of noise patterns selected by a single participant constituted the personal classification image (CI), which, when superimposed on the original base image, visualized what that participant thought a typical manager (or nursery teacher) looks like. Figure 2 depicts the group-wise CIs (generated by averaging the noise patterns within each respective condition). Because the presented stimuli are completely random, the outcome of an RCIC task is dependent on representations in the participants' mind without making any a priori assumptions about the contents of those representations (Gosselin and Schyns, 2003; Todorov et al., 2011).

#### Implicit stereotypes

After the RCIC task participants completed a variant of the Brief IAT (Sriram and Greenwald, 2009) to tap into their implicit stereotypes regarding the groups' competence. This was included for two reasons. First, we wanted to check whether the competence differences regarding the two groups were actually represented at an automatic level as well. Although previous research on visual representation has not tackled the implicitness of the extracted representation empirically, the finding that visual representations are contingent on participants' implicit prejudices (Dotsch et al., 2008) does at least allow the speculation. As a second possibility we reasoned that the strength of participants' implicit stereotypes might influence the extent to which the normative stereotype emerges in their individual CIs.

In the B-IAT, four categories (here: manager, nursery teacher, warmth, competence) are mapped on two keys, one being the focal response key, the other being the non-focal response key. At the beginning of each block, participants are instructed to react with the focal response key to all presented words belonging to one of two categories (e.g., manager and competence) and to react with the non-focal response key to all other stimuli that do not belong to one these two mentioned categories. To assess the strength with which participants associated manager and nursery teachers with the concept of competence, two different types of blocks were created. In one type of block (compatible), synonyms of competence and typical activities of managers were the focal concepts, in the other (incompatible) block the focal concepts were again synonyms of competence but this time typical activities of nursery teachers. To get a more reliable estimate, participants completed each of the blocks (20 trials) twice.

For both experimental conditions, the first block required a reaction to words that either represented competence (same words as used in the pretest) or the professional group they had previously created an image of. To represent the professional groups, verbs that described typical activities according to a pretest were chosen. In the pretest, 14 participants were asked to rank order a list of 15 activities for each occupational group according to how typical they thought this activity was for the given group. The three most typical activities were chosen as items. The category of nursery teachers was represented by the words *educate, play*, and *handicraft work*, whereas managers were represented by *delegate, lead*, and *optimize*. The non-focal stimuli in the first block referred to the other professional group or the dimension of warmth (same words as in the pretest). In the second block, competence stayed the focal category but the professional group changed. The third and fourth block mirrored the first and second. Faster responses in the compatible (blocks 1 and 3 for the manager condition; blocks 2 and 4 for the nursery teacher condition) than in the incompatible blocks were interpreted as reflecting a stronger association between manager and competence than nursery teacher and competence.

#### Explicit stereotypes

After the B-IAT participants rated both professional groups on warmth and competence with the items employed in the pretest on a 7-point-scale. Both conditions started with the profession they had created an image of and completed the ratings for the other group afterwards. Like the B-IAT, these self-reports served as manipulation checks as well as potential moderators to explore the role of individual differences.

### Image rating

The resulting CIs were judged on warmth and competence by 92 independent raters (53 women, 39 men; *M*_*age*_ = 36.22, *SD* = 14.38). Specifically, they completed the same six items as in the pretest on a 5-point-scale. Additionally, masculinity and femininity were included as rating dimensions to control for effects of gender typicality. All raters judged the group-wise images as well as 30 or 31 of the 92 individual images. Each rater rated only a subset of individual faces to keep the rating task as short as possible in order to maximize raters' concentration. Finally, the raters guessed whether the depicted person was a manager, a nursery teacher, a physician or a meter maid.

## Results

### Manipulation checks

To test whether participants in both conditions equally agreed with the normative stereotypes found in the pretest, we subjected the warmth and competence ratings for both professions to a 2 (profession) by 2 (stereotype dimension) by 2 (image classification condition) mixed-model ANOVA with the first two variables as within-subjects factors. Result revealed the predicted two-way interaction of profession and stereotype dimension, *F*_(1, 90)_ = 930.70, *p* < 0.001, η^2^_*p*_ = 0.91, not further qualified by condition, *F* < 1. Thus, across conditions we replicated the findings that nursery teachers were rated as high in warmth, *M* = 5.42, *SD* = 0.57, but low in competence, *M* = 3.25, *SD* = 0.82, whereas managers were rated as competent, *M* = 5.27, *SD* = 0.64, but low in warmth, *M* = 2.30, *SD* = 0.71. All four relevant pair-wise comparisons were significant, *p*'s < 0.001.

The difference in competence between the two groups was also reflected on an automatic level. As image classification condition was confounded with IAT block order, two separate analyses were run for the two conditions^1^. Participants in the manager condition (first block: manager and competence) were faster to map manager and competence on one response key, *M* = 859 ms, *SD* = 212 ms, than nursery teacher and competence, *M* = 1120 ms, *SD* = 349 ms, resulting in an average IAT score of *d* = 0.51, *SD* = 0.46 [internal consistency α = 0.48; for scoring algorithm see Greenwald et al. (2003)]. Participants in the nursery teacher condition (first block: nursery teacher and competence) were also faster in the blocks with manager and competence as the focal categories, *M* = 784 ms, *SD* = 147 ms, compared to the blocks with nursery teacher and competence as the focal categories, *M* = 1151 ms, *SD* = 273 ms, resulting an IAT score of *d* = 0.77, *SD* = 0.31 (α = 0.60).

Thus, we observed that participants indeed associated the two professions with the intended quadrants of the Stereotype Content Model, as indicated both by explicit ratings and the B-IAT scores. Explicit and implicit competence scores correlated in the expected direction but only moderately so (Table 1), as common for explicit-implicit correlations (Hofmann et al., 2005).

**Table 1:** **Correlations by condition of explicit stereotypes for relevant occupation group, implicit associations with competence, and independently rated warmth, competence and gender typicality of individual CIs**.

	**1**	**2**	**3**	**4**	**5**	**6**	**7**
1. Explicit warmth	−	0.08	−0.18	0.26^◦^	0.15	0.21	−0.21
2. Explicit competence	−0.02	−	0.27^◦^	0.02	−0.03	0.05	−0.16
3. Competence B-IAT	0.01	−0.09	−	−0.21	−0.13	−0.13	0.04
Average ratings of							
4. Visual warmth	0.02	0.03	0.09	−	0.69^**^	0.59^**^	0.35^*^
5. Visual competence	0.01	0.22	0.06	0.63^**^	−	0.19	0.16
6. Visual femininity	0.29^*^	0.13	0.27^◦^	0.51^**^	−0.23	−	−0.84^**^
7. Visual masculinity	0.22	0.09	−0.32^*^	−0.44^**^	0.00	−0.85^**^	−

Correlations below the diagonal for the nursery teacher condition (*n* = 46), correlations above the diagonal for the manager condition (*n* = 46). Greater B-IAT scores reflect stronger associations between competence and managers than competence and nursery teachers.

◦ p < 0.10,

* p < 0.05,

** p < 0.01.

### Image ratings per group

Next, we tested whether the internal representations revealed by the CIs also reflected the two dimensions of warmth and competence. As described earlier, independent raters, blind to the hypotheses and the origin of the images, rated the CIs on warmth, competence, masculinity, and femininity. As expected, the nursery teacher CI was rated as warmer, Cronbach's α = 0.91, *M* = 3.64, *SD* = 0.86, than the manager CI, Cronbach's α = 0.85, *M* = 2.63, *SD* = 0.80, *t*_(91)_ = 9.52, *p* < 0.001, *Cohen's d* = 1.00. More importantly, the images also differed on ratings of competence: The manager CI was rated as more competent, Cronbach's α = 0.83, *M* = 3.54, *SD* = 0.73, than the nursery teacher CI, Cronbach's α = 0.86, *M* = 2.87, *SD* = 0.70, *t*_(91)_ = 7.54, *p* < 0.001, *Cohen's d* = 0.79. Thus, both stereotype content dimensions seem to be encoded independently. To further strengthen this finding, we conducted control analyses to test whether this is indeed the case and whether the results could be more parsimoniously explained with different gender typicality (see below). As expected, the nursery teacher also looked more feminine, *M* = 3.28, *SD* = 1.08, than the manager, *M* = 1.64, *SD* = 0.75, *t*_(91)_ = 12.82, *p* < 0.001, and the manager correspondingly looked more masculine, *M* = 4.39, *SD* = 0.70, than the nursery teacher, *M* = 2.98, *SD* = 1.02, *t*_(91)_ = 11.50, *p* < 0.001. To test whether the CIs differed on both stereotype dimensions above and beyond this gender typicality we conducted within-subject regression analyses (Lorch and Myers, 1990). For each individual rater we predicted the warmth and competence ratings of the group-wise as well as the individual CIs^2^ from their femininity and masculinity ratings and saved the residuals as the remaining variance that was not explained by gender typicality. On average, the rated warmth of the images was indeed contingent on judgments of femininity, *M*_ß_ = 0.38, *SD*_ß_ = 0.31, *t*_(88)_ = 11.81, *p* < 0.001, and masculinity, *M*_ß_ = −0.08, *SD*_ß_ = 0.29, *t*_(89)_ = 2.62, *p* = 0.01. Nevertheless, the remaining variance that was not due to gender typicality differences still differentiated between the nursery teacher CI, *M*_res_ = 0.74, *SD*_res_ = 0.82 and manager CI, *M*_res_ = 0.48, *SD*_res_ = 0.86, *t*_(89)_ = 2.25, *p* = 0.03. Parallel analyses for the competence ratings yielded that ratings of competence were positively related to ratings of femininity, *M*_ß_ = 0.08, *SD*_ß_ = 0.33, *t*_(88)_ = 2.21, *p* = 0.03, as well as masculinity, *M*_ß_ = 0.25, *SD*_ß_ = 0.31, *t*_(89)_ = 7.61, *p* < 0.001. However, the variance in competence ratings that was not due to gender typicality clearly showed that the manager CI was seen as more competent, *M*_res_ = 0.75, *SD*_res_ = 0.87, than the nursery teacher CI, *M*_res_ = 0.02, *SD*_res_ = 0.86, *t*_(89)_ = −6.26, *p* < 0.001, over and above its more masculine and less feminine appearance.

**Figure 3:**
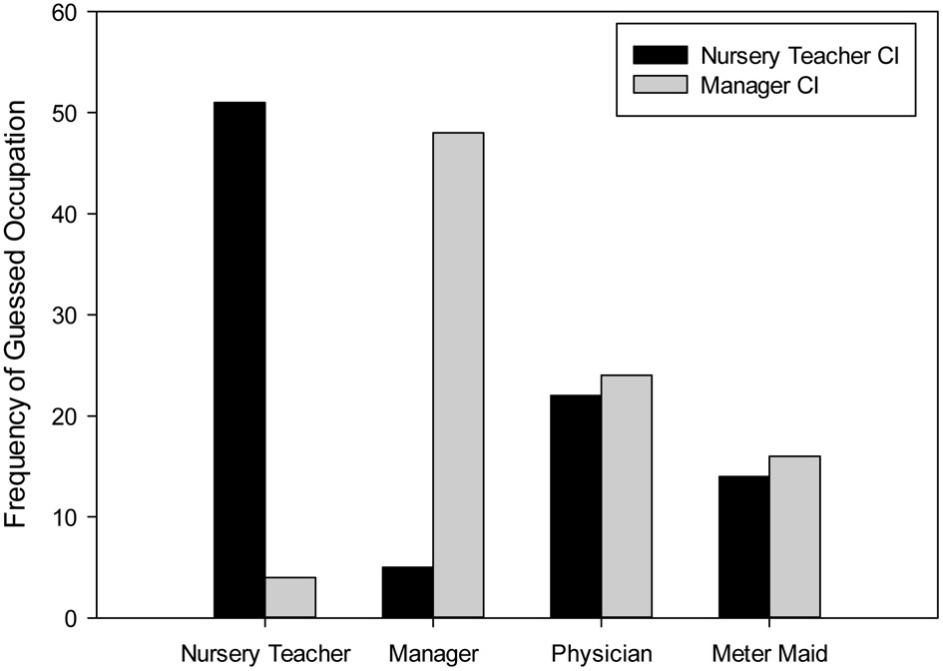
**Frequency of assumed occupation for both group-wise CIs**.

As a final task, raters were asked to guess which occupation each of the two group-wise images had. As can be seen in Figure 3, for each of the two CIs, raters disproportionally often guessed the intended occupation. The distribution of guessed occupations was significantly different from an equal distribution for both the nursery teacher CI, χ^2^_(3)_ = 51.74, *p* < 0.001, as well as the manager CI, χ^2^_(3)_ = 45.04, *p* < 0.001. To estimate which information raters used to make a correct guess we conducted binary logistic regression analyses for both CIs. We recoded the occupation guess as either correct (1) or incorrect (0) and entered warmth, competence, femininity and masculinity ratings as predictors. For the nursery teacher CI, the only significant predictor was the competence rating, *B* = −1.37, *SE* = 0.45, *Exp(B)* = 0.25, *p* = 0.002, indicating that low ratings of competence predicted the correct identification of nursery teacher. Neither the warmth information nor the two gender typicality ratings had any additional influence. Parallel analyses for the manager CI revealed that again competence ratings were a significant predictor, *B* = 0.67, *SE* = 0.32, *Exp(B)* = 1.94, *p* = 0.04, indicating that high ratings of competence predicted correct identification as manager. Again, no other predictor reached conventional levels of significance although warmth was a marginally significant negative predictor,*B* = −0.62, *SE* = 0.32, *Exp(B)* = 0.54, *p* = 0.05. Thus, the correct identification of the occupation was contingent primarily on the degree of decoded competence and not on gender typicality.

### Image ratings per individual

In addition to the group-wise CIs we computed individual CIs for each participant who completed the RCIC task. These individual images were also rated on warmth, competence, femininity, and masculinity by the same raters as the group images. However, to keep the rating task within a reasonable time and effort range, the 92 individual images were split in three subsets of 30 or 31 images. Each rater rated only the group-wise images as well as one subset of the individual images. These individual images allowed us to test the boundary conditions of the current design. First, we tested whether the group effects reported above would also show on ratings of individual images. As a second step we explored whether the degree of encoded stereotypes in these individual images would be contingent on encoders' explicitly or implicitly endorsed stereotypes.

**Figure 4:**
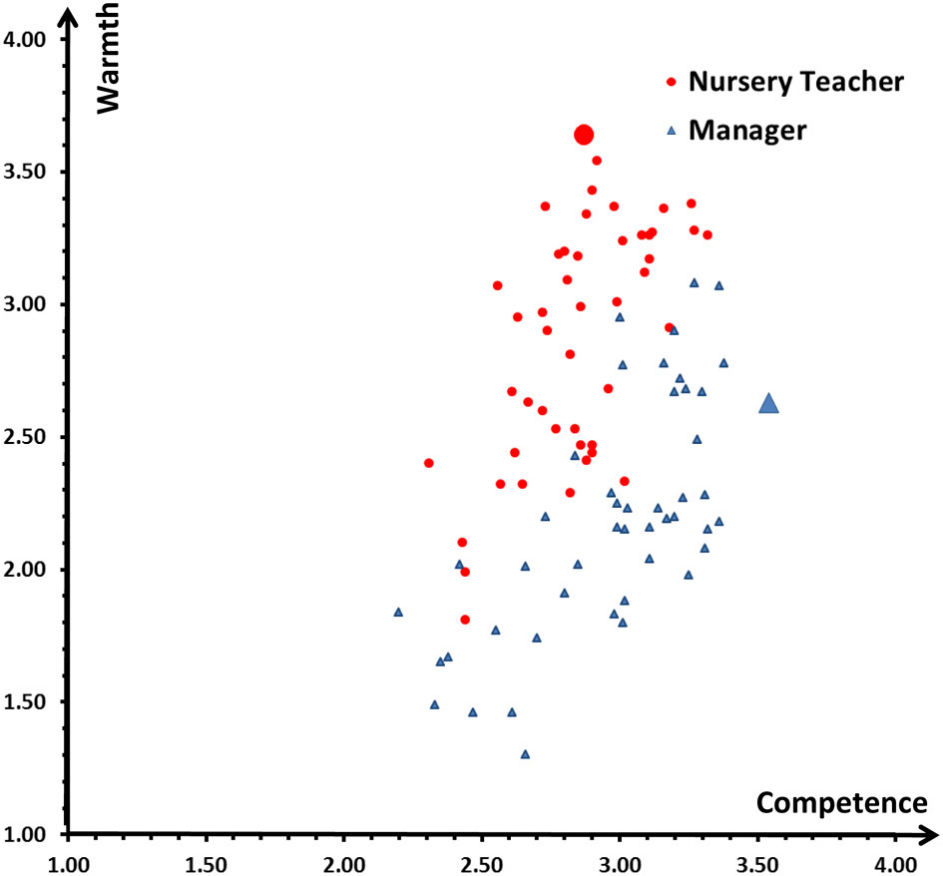
**Warmth and competence ratings (mean scores) for 92 individual classification images (small symbols) and two group-wise images (large symbols)**.

Raters showed considerable agreement in their ratings of warmth (*ICC*s between 0.94 and 0.96), competence (*ICC*s between 0.81 and 0.83), femininity (*ICC*s between 0.92 and 0.95), and masculinity (*ICC*s between 0.90 and 0.92). Ratings of each individual CI were then averaged across raters (see Figure 4 for scores of warmth and competence). We conducted four *t*-tests to test for effects of the RCIC instruction on the four rated dimensions. Paralleling the results for group-wise CIs, individual images of participants in the nursery condition were on average judged as warmer (nursery teacher: *M* = 2.85, *SD* = 0.44, manager: *M* = 2.19, *SD* = 0.44; *t*_(90)_ = 7.16, *p* < 0.001), less competent (nursery teacher: *M* = 2.85, *SD* = 0.23, manager: *M* = 2.97, *SD* = 0.32; *t*_(82, 12)_ = −2.07, *p* = 0.04), more feminine (nursery teacher: *M* = 2.61, *SD* = 0.41, manager: *M* = 1.87, *SD* = 0.29; *t*_(90)_ = 10.12, *p* < 0.001), and less masculine (nursery teacher: *M* = 3.43, *SD* = 0.31, manager: *M* = 4.06, *SD* = 0.27; *t*_(90)_ = −10.31, *p* < 0.001), than individual images of participants in the manager condition. As illustrated in Figure 4, these effects are not due to a few outliers but the distributions of individual CIs per condition hardly overlap. However, also apparent in Figure 4, the effect for the aggregated group images is generally clearer than the average effect for the individual images. One reason for this might lie in the fact that individual images are commonly much noisier and less clear.

As a next step we correlated these independent CI ratings with individual differences in the explicit and implicit endorsement of the corresponding stereotypes by the original RCIC task participants. To de-confound individual differences and the effect of the experimental condition, these correlations were conducted separately for each condition (Table 1). Overall, image ratings were largely unrelated to individual differences. As one of few exceptions, the images of participants in the nursery teacher condition who had a particular warm stereotype of nursery teachers looked more feminine to the raters. Participants in that condition who had a much stronger association of competence with managers than with nursery teachers created an image of a nursery teacher that was judged as less masculine and (marginally) more feminine by independent raters. In the manager condition, explicit ratings of warmth were marginally related to more warm-looking CIs. Given the large number of potential correlations and the problem of alpha inflation it can be concluded that surprisingly few individual differences in explicit and implicit stereotypes were reflected in individual differences in CIs.

### Control analyses to assess objective competence and warmth

To maximize differences on both dimensions, in the pretest, we selected groups that clearly differed on both dimensions, resulting in a negative correlation between warmth and competence in our design. This raises the theoretical possibility that facial cues of warmth were used to infer the inverse degree of competence. This resembles the innuendo process described for verbal information: Describing someone as particularly friendly can communicate the information that this person is not competent and vice versa (Kervyn et al., 2012).

**Figure 5:**
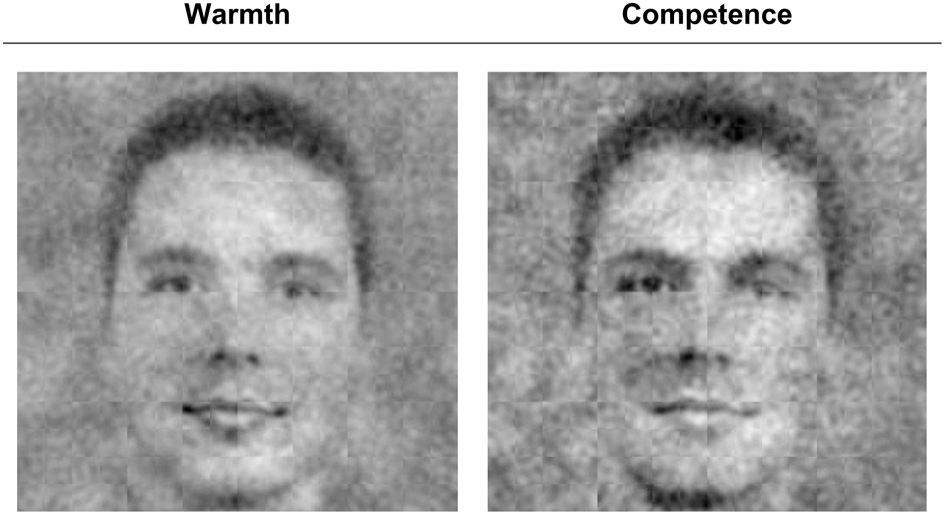
**Control classification images for warmth and competence by three hypotheses-blind participants**.

To control for this, three additional participants (2 female, 1 male; age range 23–27) created warmth and competence CIs using the same RCIC procedure as described above (Figure 5). The resulting CIs' similarity can be objectively assessed by correlating pixel gray scale values across all 262,144 pixels (512 × 512) of the noise patterns. Each pixel's value can vary between 0 (black) and 255 (white) and the zero-order correlations between the serialized pixels of two images (Table 2) reflect the extent to which these gray scale values are similar. To estimate the unique contribution of warmth and competence to the manager and nursery teacher CIs we entered the gray scale values of the warmth and competence CIs as simultaneous predictors of the corresponding values for the manager and nursery teacher CIs in two separate regression analyses. Results showed that both CIs were independently predicted by the warmth and competence CIs. The nursery teacher CI was primarily predicted by the warmth CI, ß = 0.53 (competence CI: ß = 0.12), *R*^2^ = 0.34, all *p*'s < 0.001, reflecting that the nursery teacher CI looked primarily warm and (to a much less extent) somewhat competent. In contrast, the manager CI was primarily predicted by the competence CI, ß = 0.55 (warmth CI: ß = −0.09), *R*^2^ = 0.28, all *p*'s < 0.001, providing further evidence for the notion that the manager CI looked primarily competent, and (to a lesser extent) rather cold than warm. The fact that the manager was construed as competent and cold whereas the nursery teacher was seen as warm and also a little bit competent is most likely attributable to the fact that already at the stage of the pretest the group differed more on the warmth dimension than on the competence dimension. Specifically, the nursery teacher was not judged as incompetent to the same degree as the manager was judged as cold (comparison based on *z*-standardized scores, see Figure 1). Thus, both stereotype content dimensions were clearly accessed spontaneously and independently encoded visually in the CIs.

**Table 2:** **Correlation of pixel luminance values across 512 × 512 pixels**.

	**Warmth CI**	**Competence CI**	**Nursery teacher CI**
Warmth CI	–	–	–
Competence CI	0.34^***^	–	–
Nursery Teacher CI	0.57^***^	0.30^***^	–
Manager CI	0.10^***^	0.52^***^	0.04^***^

*** *p* < 0.001

## Discussion

The current work presents first evidence for the spontaneous use of the two fundamental dimensions of social judgment, warmth and competence, in visual representation of group members' faces, providing initial support for the tight connection between the big two dimensions of social perception and two important dimensions of face perception: trustworthiness (or warmth) and competence (see Todorov et al., 2005, 2013; Oosterhof and Todorov, 2008; for work that models dimensions of face evaluation, focusing specifically on trustworthiness and competence). People seem to use facial cues indicative of both dimensions to make sense of social groups in a parsimonious, non-verbal and spontaneous manner.

This was observed at the level of an aggregated representation of a shared stereotype (nursery teachers as warm but incompetent; managers as cold but competent) as well as at the individual level. However, individual differences in the degree to which visual representations of the respective group were judged as either warm or competent were mostly unrelated to individual differences in the degree to which these stereotypes were endorsed either on an explicit level or (for competence) at an implicit level. Although it is conceivable that visual representation and verbal representation function largely independent, a more parsimonious explanation could be based on the fact that we purposefully chose socially shared stereotypes at the potential cost of restricting meaningful inter-individual variance. Another possibility lies in the method: individual CIs are commonly noisier than group-wise CIs. Thus, individual images may have been too noisy for raters to decode the inter-individually different information encoded in them. A third explanation is that the stimuli employed for the profession categories (e.g., *play* for nursery teachers vs. *lead* for managers) in the B-IAT might have differed intrinsically on implied competence, thereby masking individual differences in associations between competence and the profession categories.

Nevertheless, the current work is the first to systematically test whether both dimensions of the SCM are encoded in and decoded from visual representations of group member's faces. Above we have shortly mentioned the critical possibility of an innuendo effect. It might be conceivable that in our study encoders merely created warm nursery teachers and cold manager and that later raters used this decoded warmth information to infer competence in a compensatory manner. A warm-looking person is probably not competent and vice versa. One way to address this possibility would be to have visual representations of all four SCM quadrants to test the expected differences in a more complete manner. We have chosen a different approach. By having independently created prototypical images of what a warm person looks like and what a competent person looks like we were able to address this possibility by calculating objective similarities between these images and group-wise CIs. An innuendo effect could have been demonstrated if the nursery teacher CI would have been similar to the warmth CI (positive correlation), the manager CI would have been dissimilar to the warmth CI (negative correlation) and both would have been unrelated to the competence CI. However, this pattern was not observed in our data. Instead, the manager CI was clearly more similar to the competence CI than to the warmth CI and vice versa for the nursery teacher CI. We conclude that both SCM dimensions were encoded in the CIs.

Although our data convincingly show that both stereotype dimensions were visually encoded, it is still conceivable that an effect of warmth primacy would be reflected in larger effects. The mean differences between nursery teachers and managers were indeed somewhat larger for the warmth dimension than for the competence dimensions but this could be attributable to the fact that in the pretest as well in the manipulation check of explicit stereotype endorsement the same difference was found. Potentially, managers and nursery teachers simply differ more on the warmth dimension than the competence dimension. Given this, it seems even more surprising that the dimension that predicted successful detection of the occupation was competence. Raters who judged the nursery teacher as particular incompetent and the manager as particular competent were more likely to correctly identify or guess their occupation. It thus seems that facial cues of competence sometimes bear more weight than facial cues of warmth, speaking against a general primacy of warmth information.

We argue that both warmth and competence are encoded in a facial visual mental representation. However, although tempting, one should take care with equating the CIs with actual visual representations residing in participants' cognitive system. First, although the CIs are functions of mental representations, they are also functions of the set of noise patterns used, the employed base image, motivation of participants, etc. Second, although the CIs are meaningful, participants might not actually have visual mental representations of managers or nursery teachers in their minds. For instance, participants might resort to verbal knowledge about managers (i.e., that they are competent) and use their visual representation of competent faces as proxy to make their decisions in the RCIC task. Third, just because the task is of a visual nature, the assessed representations are not necessarily visual [for an analogous discussion of the latter two points, see Dotsch et al. (2013)].

However, we believe that the most parsimonious and therefore, most likely explanation for our data is that the CI is tapping into visual representations of managers and nursery teachers. First, it is safe to assume that our participants have had visual experience with both groups, a necessary prerequisite for the existence of visual mental representations of both groups. If visual information about typical members of the respective groups is available, it is very unlikely that the cognitive system should instead use non-visual information to complete the RCIC task. Second, participants did not *have* to use warmth and competence information in the RCIC task (the concepts of warmth and competence were not mentioned at all prior to and during the task). The fact that the CIs did include visual cues diagnostic of warmth and competence in our view is therefore, first evidence of *spontaneous* encoding of dimensions of warmth and competence in *visual* mental representations of faces (i.e., visual stereotypes).

### Conflict of interest statement

The authors declare that the research was conducted in the absence of any commercial or financial relationships that could be construed as a potential conflict of interest.

## Footnotes

^1^This confound and the fact that we did not include an equivalent measure of automatic warmth stereotypes could be seen as a liability of the current design. We thus re-recruited 50 of the participants for a second lab session approximately 3 months after the first date (23 from the original manager condition and 27 from the original nursery teacher condition). They only completed a B-IAT in a fixed order (details available upon request). Results showed a stronger implicit association of warmth with nursery teachers than managers, *IATd* = −0.65, and a stronger association of competence with managers than nursery teachers, *IATd* = 0.65. Both effects were independent of the image classification condition in the original experiment. It is thus safe to assume that the participants indeed automatically associated both professions with the two stereotype dimensions to a comparable degree and the obtained results are not due to block order. Full analyses are obtainable from the authors.

^2^As in these intra-individual regressions, rating targets (i.e., classification images) are treated as cases, a restriction to the group-wise CIs would have resulted in only two cases per regression. No regression could be calculated for participants who always gave the same rating on either the predictors or the criterion in any of the analyses, resulting in 89 cases for the warmth analyses and 90 cases for the competence analyses.
